# The Genetic Background and Culture Medium Only Marginally Affect the In Vitro Evolution of *Pseudomonas aeruginosa* Toward Colistin Resistance

**DOI:** 10.3390/antibiotics14060601

**Published:** 2025-06-13

**Authors:** Matteo Cervoni, Antonio Maria Ferriero, Alessandra Lo Sciuto, Francesca Guidi, Naida Babić Jordamović, Silvano Piazza, Olivier Jousson, Alfonso Esposito, Francesco Imperi

**Affiliations:** 1 Department of Science, University Roma Tre, 00146 Rome, Italy; matteo.cervoni@uniroma3.it (M.C.); ant.ferriero@stud.uniroma3.it (A.M.F.); alessandra.losciuto@uniroma3.it (A.L.S.); fra.guidi1@stud.uniroma3.it (F.G.); 2 International Centre for Genetic Engineering and Biotechnology, 34149 Trieste, Italy; naida.jordamovic@icgeb.org (N.B.J.); silvano.piazza@icgeb.org (S.P.); alfonso.esposito@univr.it (A.E.); 3 Department of Cellular, Computational and Integrative Biology, University of Trento, 38123 Trento, Italy; olivier.jousson@unitn.it; 4 Department of Biotechnology, University of Verona, 37134 Verona, Italy; 5 IRCCS Fondazione Santa Lucia, 00179 Rome, Italy; 6 NBFC—National Biodiversity Future Center, 90121 Palermo, Italy

**Keywords:** artificial sputum medium, colistin, fatty acids, human serum, in vitro evolution, norspermidine, lipid A, PA14, PAO1, polymyxins

## Abstract

**Background/Objectives:** Colistin is a last-resort treatment for *Pseudomonas aeruginosa* multidrug-resistant infections, but resistance to it is emerging. While colistin resistance in *P. aeruginosa* is typically associated with chromosomal mutations inducing lipopolysaccharide (LPS) aminoarabinosylation, other mutations unrelated to LPS modifications have been proposed to influence the extent of colistin resistance. Here, we examined whether the genetic background and culture conditions affect the evolution of high-level colistin resistance in this bacterium. **Methods**: We performed in vitro evolution experiments in the presence or absence of increasing colistin concentrations with two phylogenetically distant reference strains in a standard laboratory medium and in two media mimicking *P. aeruginosa* growth during lung or systemic infections. Resistance-associated mutations were identified by comparative genomics, and the role of selected mutated genes was validated by allele replacement, deletion, or conditional mutagenesis. **Results**: Most colistin-resistant mutants carried mutations in genes belonging to four functional groups: two-component systems controlling LPS aminoarabinosylation (PmrAB, PhoPQ), LPS biosynthesis, the production of the polyamine norspermidine, and fatty acid metabolism. No mutation was exclusively and invariably associated with a specific strain or medium. We demonstrated that norspermidine is detrimental to the acquisition of colistin resistance upon PmrAB activation and that impaired fatty acid biosynthesis can promote colistin resistance, even if it increases susceptibility to other antibiotics. **Conclusions**: The evolution of colistin resistance in *P. aeruginosa* appeared to be only marginally affected by the genetic background and culture conditions. Notably, mutations in fatty acid biosynthetic genes represent a newly identified genetic determinant of *P. aeruginosa* colistin resistance, warranting further investigation in clinical isolates.

## 1. Introduction

The rise in antibiotic resistance in bacterial pathogens, particularly among Gram-negative bacteria, represents an alarming global threat [1]. In a landscape of limited therapeutic options, colistin, a cationic antibiotic belonging to the polymyxin family, has emerged as one of the last-line antibiotics for the treatment of infections caused by multidrug-resistant Gram-negative bacteria [2]. However, the reintroduction of colistin into clinical practice has led to the emergence and spread of resistant isolates, challenging healthcare professionals with ever-more pressing issues [3,4].

Colistin is a decapeptide consisting of a seven-member cyclic ring and a tripeptide side chain bound to a short fatty acid. It exerts its antibacterial activity by interacting with the anionic lipid A moiety of lipopolysaccharide (LPS) in the outer membrane of Gram-negative bacteria [5]. This interaction displaces divalent cations, such as Ca^2+^ and Mg^2+^, destabilizing the LPS layer and causing outer-membrane instability and cell death [3,5]. Recently, it has been demonstrated that colistin causes the perturbation of the cytoplasmic membrane and cell lysis by also binding to newly synthesized LPS molecules transiently present in the periplasmic leaflet of the cytoplasmic membrane [6]. The primary mechanisms of colistin resistance in most Gram-negative bacteria involve the remodeling of lipid A through the covalent addition of positively charged molecules, such as 4-amino-4-deoxy-L-arabinose (L-Ara4N) and phosphoethanolamine (PEtN). This reduces the negative charge of lipid A, leading to a decreased affinity of polymyxins for the LPS layer [7,8].

*Pseudomonas aeruginosa* is an opportunistic Gram-negative pathogen causing severe infections in immunocompromised individuals and patients with cystic fibrosis [9]. In this bacterium, colistin resistance is generally associated with the induction of the *arnBCADTEF-ugd* (*arn*) operon [7,10]. This operon encodes the enzymes responsible for L-Ara4N-dependent modification of lipid A and is regulated by a complex network involving several two-component systems (TCSs), among which PhoPQ and PmrAB play a major role in controlling *arn* gene expression [7,11]. Indeed, mutations in the genes encoding these TCSs that result in the constitutive activation of the *arn* operon are usually identified in *P. aeruginosa* colistin-resistant clinical isolates [12,13,14].

Several experimental evolution studies have been conducted to investigate the genetic determinants underlying the acquisition of colistin resistance in *P. aeruginosa* [15,16,17,18,19,20,21,22]. These studies confirmed the crucial role of lipid A aminoarabinosylation for the acquisition of colistin resistance in *P. aeruginosa*, as L-Ara4N deficient mutants were found to be unable to evolve colistin resistance [15,16] and amino acid substitutions in the TCSs PmrAB and/or PhoPQ were invariably found in colistin-resistant mutants [15,17,18,19]. However, it has been demonstrated that lipid A aminoarabinosylation alone is not sufficient to confer high levels of colistin resistance and that its impact on resistance varies significantly among different strains and culture conditions [20]. Indeed, the evolution toward high-level colistin resistance seems to be a complex, multistage process that also involves mutations in genes not directly correlated with lipid A modifications [15,19,20,21,22]. Various studies, employing different experimental evolution approaches, culture conditions, and/or strains, have identified mutations in different genes besides TCS-encoding genes; however, the specific mutations varied considerably across studies [15,17,18,19]. So far, a clear and unique evolutionary pathway for acquiring high-level colistin resistance has not been identified in *P. aeruginosa*.

The aim of the present study was to investigate whether the evolutionary trajectories leading to high-level colistin resistance in *P. aeruginosa* are influenced by the genetic background and/or the growth conditions. To address this issue, we experimentally evolved the two reference strains PAO1 and PA14, which are phylogenetically distant and representative of the two major *P. aeruginosa* lineages [23], toward colistin resistance in three different media, including media that should mimic the growth of *P. aeruginosa* during pulmonary or systemic infection. Next, allele replacement, deletion, and conditional mutagenesis were performed to assess the actual contribution of selected colistin-associated mutations to colistin resistance in *P. aeruginosa*.

## 2. Results

### 2.1. Experimental Evolution of High-Level Colistin Resistance in Different Media

**Figure 1:**
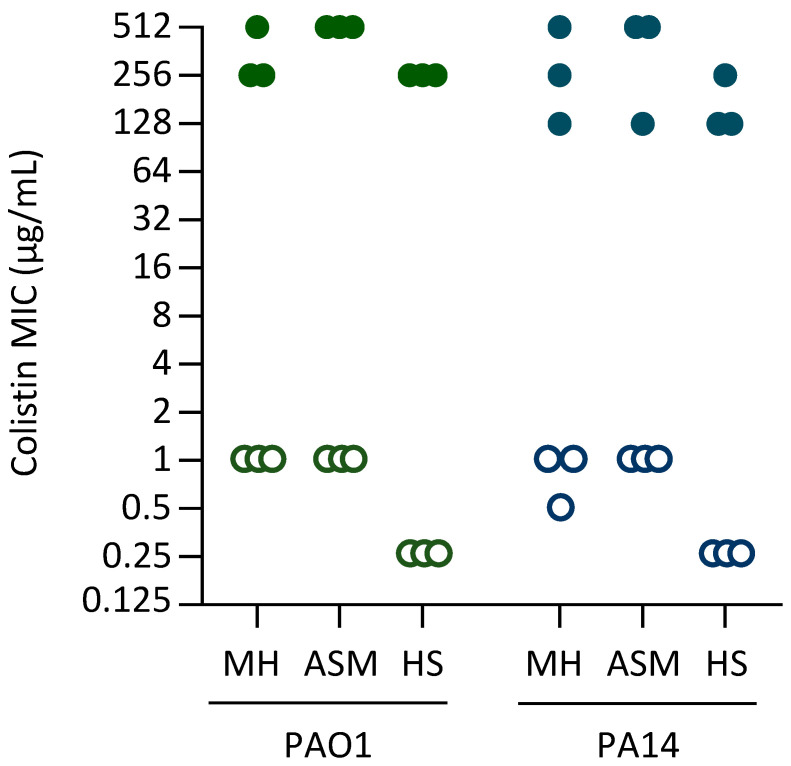
Colistin MIC of the clones evolved in MH, ASM, or HS (as indicated in the figure) in the presence of colistin (filled symbols) and the control strains evolved for the same number of passages in the absence of colistin (empty symbols). MIC was determined after 20 h of growth in MH and represents the mode of at least three independent assays. The MIC for the parental strains PAO1 and PA14 was 0.25 μg/mL (not shown in the figure).

To investigate whether different genetic backgrounds or culture conditions could influence the evolutionary pathways driving the acquisition of high levels of colistin resistance in *P. aeruginosa*, three independent cultures of two different reference strains (PAO1 and PA14) were sequentially cultured in the presence of increasing concentrations of colistin (up to 256 µg/mL) in three different culture media: the standard laboratory medium Mueller–Hinton (MH), artificial sputum medium (ASM), and heat-inactivated human serum (HS) (Figure S1). The latter two media were used to mimic, at least partially, the growth conditions that *P. aeruginosa* faces during pulmonary or systemic infections. In parallel, three independent cultures for each strain and medium were cultured for the same number of passages in the absence of colistin, as a control to identify mutations that could emerge during adaptation to each specific culture condition, thus unrelated to colistin resistance. MIC assays confirmed that clones evolved in the presence of colistin acquired high levels of resistance (colistin MIC = 128–512 µg/mL), while control clones evolved in media without colistin did not show any increase in colistin resistance as compared to the parental strains (Figure 1).

### 2.2. Genes Mutated in Colistin-Resistant Clones

A comparative genomic analysis of clones evolved in the presence or absence of colistin and the corresponding parental strains revealed the presence of several mutations exclusively found in colistin-resistant clones. Each colistin-resistant clone carried two to seven different mutations in coding sequences, many of which were specific to each clone (Table S1). Genes or pathways that were found to be mutated in at least three independent resistant clones are shown in Table 1. Recurring mutations associated with colistin resistance were observed in genes involved in four functional categories, namely, the regulation of genes involved in lipid A modification, the synthesis or modification of LPS, the production of the polyamine norspermidine, and fatty acid biosynthesis.

Overall, our findings align with previous studies that have linked some of these genes to polymyxin resistance in *P. aeruginosa* [15,17,18,19,24], with the exception of fatty acid biosynthetic genes, which were not previously associated with polymyxin resistance. However, a metabolomic study reported a reduction in phospholipid levels in polymyxin-resistant mutants of *P. aeruginosa* compared to the polymyxin-sensitive parental strain, suggesting that decreased phospholipid production may play a role in polymyxin resistance [25]. Notably, none of these mutations were exclusively and invariably associated with a specific medium or bacterial strain (Table 1). Thus, contrary to our original hypothesis inspired by data from the literature, this result suggests that the culture conditions and/or the genetic background do not have a relevant effect on the evolutionary trajectories followed by *P. aeruginosa* in the acquisition of colistin resistance. In all colistin-evolved mutants, at least one mutation was observed in the TCS genes *pmrAB* or *phoPQ*, confirming the crucial role of TCSs that control the expression of the *arn* operon in the acquisition of colistin resistance in *P. aeruginosa* [7,11,15]. The highest number of mutations was found in the *pmrB* gene (11 out of 18 colistin-resistant clones), followed by mutations in *phoQ* (5 out of 18 colistin-resistant clones), encoding the sensor kinases of the TCSs PmrAB and PhoPQ, respectively (Table 1). Notably, only one or two mutations were found in the cognate response regulator genes *phoP* and *pmrA*, respectively (Table 1), indicating that the sensor kinase genes are more prone to acquiring mutations conferring resistance. Notably, while all mutations in *pmrB* were nonsynonymous, except for a 9 bp in-frame deletion in the clone PA14 ColR ASM3, 60% of *phoQ* mutations were frameshifts, deletions, or a gene disruption due to a genomic rearrangement (Table S1). This is in line with the mode of action of *P. aeruginosa* PhoQ, which primarily dephosphorylates and, thus, inactivates its response regulator PhoP [26,27]. Accordingly, loss-of-function mutations and/or deletions in *phoQ* were reported to increase polymyxin resistance in this bacterium [28,29].

Besides the TCSs controlling *arn* gene expression, several colistin-resistant clones carried missense mutations in genes involved in LPS or fatty acid biosynthesis, as well as frameshift or nonsense mutations in genes responsible for norspermidine production (Table 1 and Table S1). As mentioned above, mutations in fatty acid biosynthetic genes were not previously identified in colistin-resistant *P. aeruginosa* strains. Moreover, although mutations in LPS and norspermidine genes had previously been associated with colistin resistance [15,19,21,22,24], the mechanisms by which these pathways affect resistance had remained unexplored.

To investigate this issue, we generated deletion or conditional mutants in norspermidine, fatty acid, or LPS genes in different genetic backgrounds, i.e., the parental strains (PAO1 and PA14), derivatives that constitutively express the enzymes for lipid A aminoarabinosylation (P*rpsA*::*arn* recombinant strains) [20], and derivatives carrying a mutated *pmrB* allele (*pmrB^M292T^*), which was found in one of our in vitro-evolved colistin-resistant mutants (PAO1 Col^R^ MH3) and was also previously identified in polymyxin-resistant *P. aeruginosa* isolates [19,30]. This was carried out to investigate whether the impact on colistin resistance of the genes frequently mutated in in vitro-evolved colistin-resistant clones may be influenced by lipid A aminoarabinosylation and/or the induction of the PmrAB regulon. Notably, mutations in *bamA* and *pitA*, previously associated with colistin resistance in *P. aeruginosa* [15,19], were also observed in a few colistin-resistant clones (Table S1) but were not further investigated in this study.

As expected, the replacement of *pmrB* with the *pmrB^M292T^* allele promoted colistin resistance, although with a strain-dependent effect. Specifically, the *pmrB^M292T^* allele increased the colistin MIC by 4-fold in PAO1 and 16-fold in PA14 (Figure S2). Notably, the increase in the colistin MIC due to the *pmrB^M292T^* allele was higher than that caused by the expression of *arn* genes only (P*rpsA*::*arn* strains) (Figure S2). This could reasonably be due to the fact that, in addition to the *arn* operon, PmrAB controls directly or indirectly the transcription of many genes involved in various cellular pathways, including cell envelope homeostasis [11,24,31].

### 2.3. Norspermidine Has a Negative Impact on Colistin Resistance in P. aeruginosa

**Figure 2:**
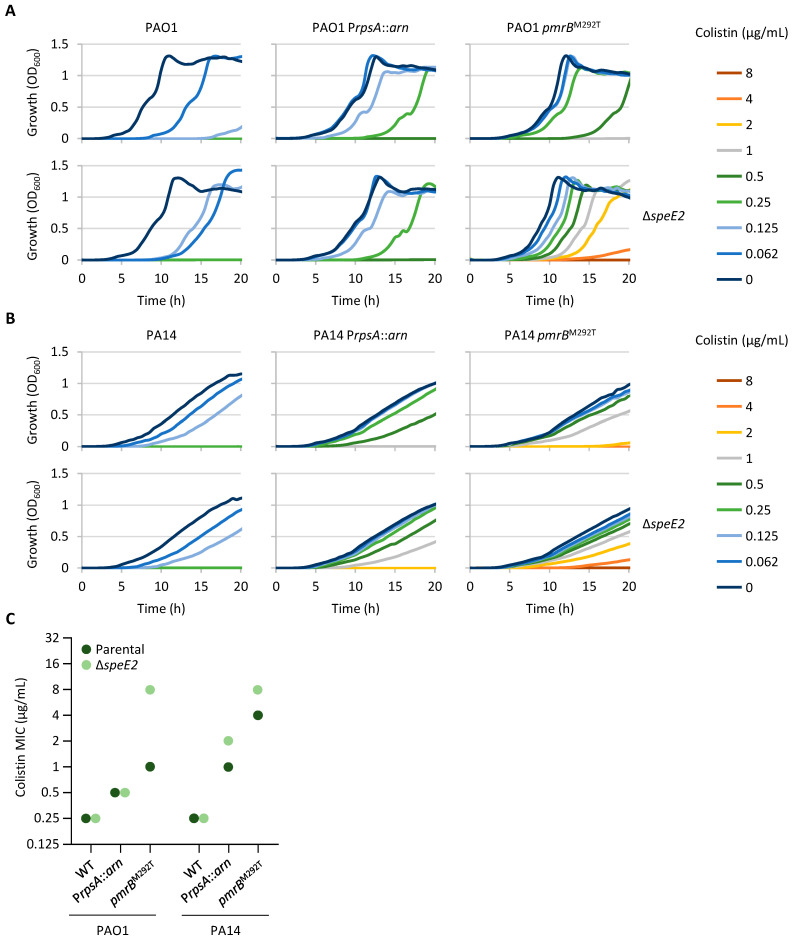
Effect of norspermidine on growth and colistin resistance. (**A**) Growth curves of PAO1, PAO1 P*rpsA*::*arn*, PAO1 *pmrB^M292T^*, PAO1 Δ*speE2*, PAO1 P*rpsA*::*arn* Δ*speE2*, and PAO1 *pmrB^M292T^* Δ*speE2* in MH supplemented with increasing colistin concentrations. (**B**) Growth curves of PA14, PA14 P*rpsA*::*arn*, PA14 *pmrB^M292T^*, PA14 Δ*speE2*, PA14 P*rpsA*::*arn* Δ*speE2*, and PA14 *pmrB^M292T^* Δ*speE2* in MH supplemented with increasing colistin concentrations. (**C**) Colistin MIC for the above-mentioned strains after 20 h of growth in MH. Growth curves are representative of at least three independent experiments giving similar results. MIC values correspond to the mode of at least three independent experiments.

The genes *speD2* and *speE2* encode the S-adenosylmethionine decarboxylase and aminopropyltransferase responsible for norspermidine synthesis [31]. These genes are located upstream of *pmrAB* and belong to the PmrAB regulon [11,31]. Several colistin-resistant clones acquired nonsense or frameshift mutations in *speD2* or *speE2* (Table 1 and Table S1), suggesting a possible negative impact of norspermidine on colistin resistance. To investigate this hypothesis, *speE2* deletion mutants were generated in the previously mentioned *P. aeruginosa* strains (PAO1, PAO1 P*rpsA*::*arn*, PAO1 *pmrB^M292T^*, PA14, PA14 P*rpsA*::*arn*, and PA14 *pmrB^M292T^*). All Δ*speE2* mutants exhibited a growth profile similar to the parental strains in the absence of colistin (Figure 2A,B), indicating that norspermidine is not required for growth under the experimental conditions tested in this study. The deletion of *speE2* in the wild-type strains PAO1 and PA14 did not increase the colistin MIC, although growth in the presence of colistin at a 0.5×MIC was markedly improved in the PAO1 Δ*speE2* mutant compared to the wild-type strain (Figure 2A). On the other hand, *speE2* deletion increased the colistin MIC in both strains carrying the *pmrB^M292T^* allele, with a much higher increase in PAO1 than in PA14 (eight- vs. two-fold increase compared to the parental *pmrB^M292T^* strains, respectively) (Figure 2C). Notably, the colistin MIC was identical (8 μg/mL) for PAO1 *pmrB^M292T^* Δ*speE2* and PA14 *pmrB^M292T^* Δ*speE2*, suggesting that the diverse effect of *speE2* deletion could be due to the different impacts of the *pmrB^M292T^* allele on colistin resistance in the two strains. Finally, the inactivation of *speE2* did not affect colistin resistance in PAO1 P*rpsA*::*arn*, while it led to a two-fold increase in the colistin MIC in the equivalent PA14 strain (Figure 2C), highlighting another difference between the PAO1 and PA14 reference strains.

### 2.4. Impaired Expression of an LPS Biosynthetic Gene Has No Impact on Colistin Resistance

Missense mutations in three lipid A biosynthetic genes (*lpxA*, *lpxC*, and *lpxD*) were identified in six colistin-resistant clones. Moreover, missense mutations in *lpxO2* were found in four mutants, while one mutant carried a frameshift mutation in *lpxT* (Table 1 and Table S1). LpxA, LpxC, and LpxD catalyze the first three steps of lipid A biosynthesis [32] and, as expected, are essential in *P. aeruginosa* [33,34,35], while the LpxO2 and LpxT enzymes are responsible for lipid A modification through the hydroxylation of a secondary acyl chain or the phosphorylation of 1 or 4′ phosphate groups of lipid A, respectively [36,37]. These lipid A modifications are not essential in *P. aeruginosa* and, according to previous evidence, do not appear to affect colistin resistance in this bacterium [36,37,38]. In contrast, missense mutations in LPS biosynthetic genes were previously observed in colistin-resistant *P. aeruginosa* strains [15,18], suggesting that changes in Lpx enzyme functionality might play a role in the acquisition of colistin resistance in *P. aeruginosa*.

**Figure 3:**
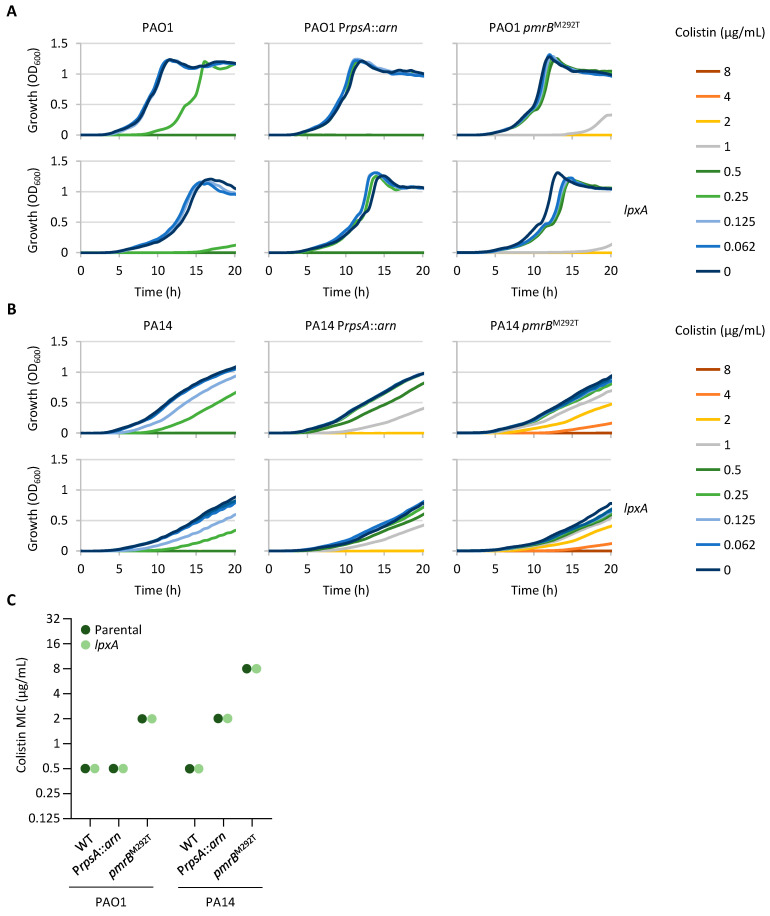
Effect of suboptimal *lpxA* gene expression on colistin resistance. (**A**) Growth curves of PAO1, PAO1 P*rpsA::arn*, PAO1 *pmrB*^M292T^, PAO1 *araC*-P_BAD_::*lpxA* Δ*lpxA*, PAO1 P*rpsA::arn araC*-P_BAD_::*lpxA* Δ*lpxA*, and PAO1 *pmrB*^M292T^ *araC*-P_BAD_::*lpxA* Δ*lpxA* in MH supplemented with 0.125% arabinose and increasing colistin concentrations. (**B**) Growth curves of PA14, PA14 P*rpsA::arn*, PA14 *pmrB*^M292T^, PA14 *araC*-P_BAD_::*lpxA* Δ*lpxA*, PA14 P*rpsA::arn araC*-P_BAD_::*lpxA* Δ*lpxA*, and PA14 *pmrB*^M292T^ *araC*-P_BAD_::*lpxA* Δ*lpxA* cultured in MH supplemented with 0.125% arabinose and increasing colistin concentrations. (**C**) Colistin MIC for the above-mentioned strains after 20 h of growth in MH supplemented with 0.125% arabinose. Growth curves are representative of at least three independent experiments giving similar results. MIC values correspond to the mode of at least three independent experiments.

We hypothesized that missense mutations in individual LPS biosynthetic genes are more likely to reduce rather than increase LPS biosynthesis rates and that impaired LPS biosynthesis could provide an advantage in the presence of colistin by reducing the amount of LPS in the outer membrane, which represents the primary target of colistin. To investigate the relationship between LPS biosynthesis rates and colistin resistance, we generated conditional mutants carrying an arabinose-inducible *lpxA* gene. As expected, the growth of the *lpxA* conditional mutants was induced by arabinose in a dose-dependent manner (Figure S3), confirming the essentiality of the *lpxA* gene in *P. aeruginosa*. To verify the hypothesis that reduced LPS biosynthesis may decrease colistin sensitivity, we performed growth and MIC assays in the presence of an arabinose concentration (0.125%) at which the growth of the *lpxA* conditional mutants was slightly impaired as compared to their parental strains (Figure S3), which is indicative of suboptimal LPS biosynthesis rates. In contrast to our hypothesis, no increase in colistin resistance was observed in the *lpxA* conditional mutants with respect to the corresponding parental strains, and the growth of the PAO1 *lpxA* conditional mutant at a 0.5×MIC of colistin was even severely impaired as compared to its wild-type strain (Figure 3). Further reducing the expression of *lpxA* by supplementing the medium with a lower arabinose concentration (0.0625%) made the *lpxA* conditional mutants more sensitive rather than more resistant to colistin (Figure S4).

These results suggest that reduced expression of the *lpxA* gene and, consequently, reduced biosynthesis of LPS do not provide an advantage in terms of colistin resistance, either in wild-type strains or in strains already carrying genetic determinants that decrease colistin susceptibility (i.e., *pmr^BM292T^* mutants and P*rpsA*::*arn* recombinant strains).

### 2.5. Compromised Fatty Acid Biosynthesis Can Contribute to Colistin Resistance

Three colistin-resistant clones carried missense mutations in fatty acid biosynthesis genes, namely, *accD*, *acpP*, *fapB*, and *fabY*, with *fapB* and *fabY* mutations present in the same mutant (Table 1 and Table S1). Fatty acids are fundamental components of phospholipids and are therefore essential for bacterial cells. Accordingly, transposon mutagenesis studies confirmed that the above-mentioned genes are all essential in *P. aeruginosa* [34,35]. To verify the impact of fatty acid biosynthesis rates on colistin resistance, we generated conditional mutants for the *accD* gene. This gene encodes a subunit of the acetyl-CoA carboxylase, which catalyzes the carboxylation of acetyl-CoA to produce malonyl-CoA, the first committed step of fatty acid biosynthesis [39]. Also in this case, the growth of all conditional mutants relied on arabinose in a dose-dependent manner (Figure S3), confirming the essentiality of the *accD* gene. However, differently from what was observed for the *lpxA* conditional mutants and other arabinose-dependent conditional mutants previously generated in our laboratory [40,41,42,43], the growth of the *accD* conditional mutants was significantly impaired even in the presence of very high arabinose concentrations (up to 2%) (Figure S3), suggesting that the *araC*-P_BAD_ regulatory element is not able to restore *accD* expression at physiological levels. To assess the impact of impaired fatty acid synthesis on colistin susceptibility, conditional mutants were cultured in the presence of 0.5% arabinose and the incubation time was extended to promote the growth of these mutants.

**Figure 4:**
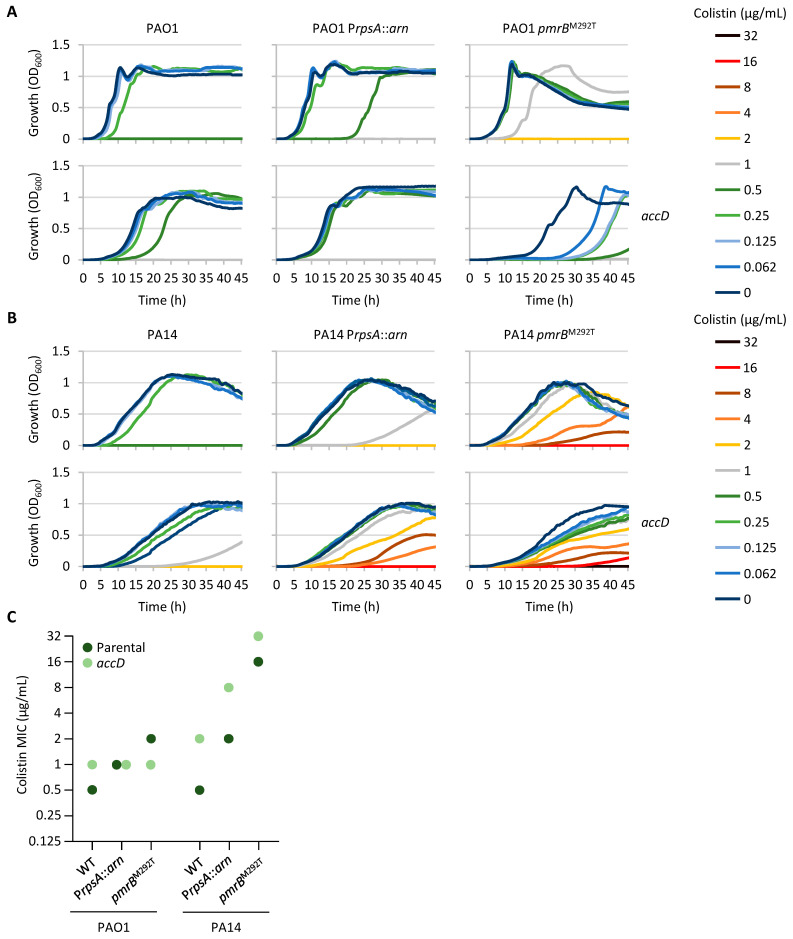
Effect of suboptimal *accD* gene expression on colistin resistance. (**A**) Growth curves of PAO1, PAO1 P*rpsA::arn*, PAO1 *pmrB*^M292T^, PAO1 *araC*-P_BAD_::*accD* Δ*accD*, PAO1 P*rpsA::arn araC*-P_BAD_::*accD* Δ*accD*, and PAO1 *pmrB*^M292T^ *araC*-P_BAD_::*accD* Δ*accD* in MH supplemented with 0.5% arabinose and increasing colistin concentrations. (**B**) Growth curves of PA14, PA14 P*rpsA::arn*, PA14 *pmrB*^M292T^, PA14 *araC*-P_BAD_::*accD* Δ*accD*, PA14 P*rpsA::arn araC*-P_BAD_::*accD* Δ*accD*, and PA14 *pmrB*^M292T^ *araC*-P_BAD_::*accD* Δ*accD* in MH supplemented with 0.5% arabinose and increasing colistin concentrations. (**C**) Colistin MIC for the above-mentioned strains after 20 h of growth in MH supplemented with 0.5% arabinose. Growth curves are representative of at least three independent experiments giving similar results. MIC values correspond to the mode of at least three independent experiments.

As shown in Figure 4, different results were obtained for the two strains PAO1 and PA14. Indeed, suboptimal expression of *accD* had no clear impact on colistin resistance in PAO1, as the colistin MIC remained the same or only showed two-fold variations (increase or decrease) between the *accD* conditional mutants and the corresponding parental strains. In contrast, all the PA14 derivatives carrying the *accD* conditional mutation showed an increase in the colistin MIC compared to the parental strains. Such an increase was more pronounced in the wild-type and P*rpsA::arn* backgrounds (four-fold), while it was lower in the strain carrying the *pmrB*^M292T^ allele (two-fold) (Figure 4). This difference may be due to the higher level of colistin resistance of PA14 *pmrB*^M292T^ compared to the other PA14 strains, which is probably more difficult to increase further. In conclusion, this experiment confirmed that suboptimal expression of the *accD* gene and, therefore, impaired fatty acid biosynthesis can increase colistin resistance in *P. aeruginosa*, although the effect appears to be strain-dependent.

### 2.6. Mutations Associated with Colistin Resistance Can Influence Sensitivity to Other Antibiotic Classes

**Figure 5:**
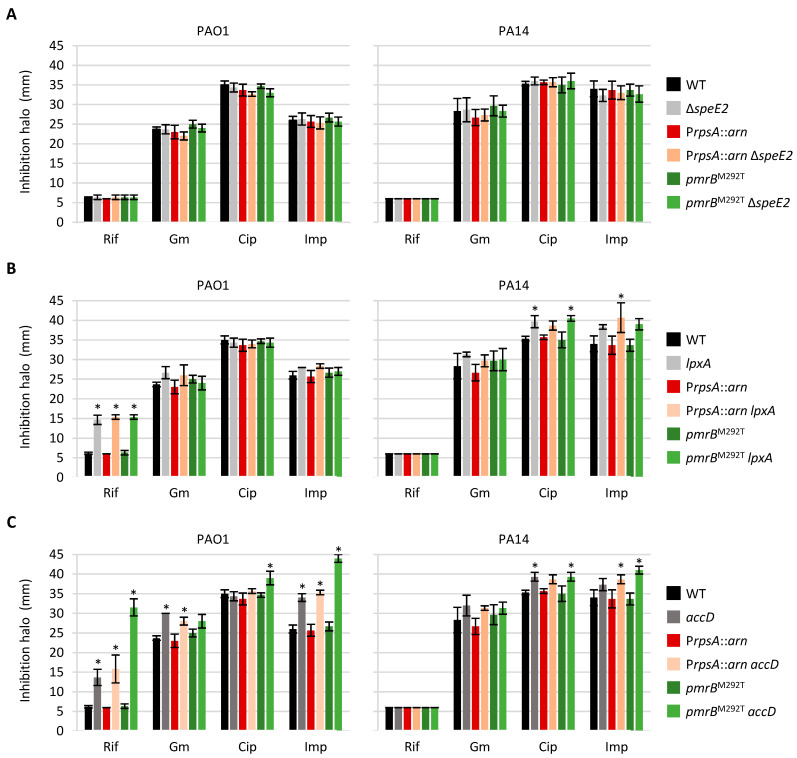
Kirby–Bauer disk diffusion assay for the *speE2* deletion mutants and the *lpxA* and *accD* conditional mutants. (**A**) Antibiotic susceptibility profile of the *speE2* deletion mutants and their parental strains on MH agar plates. (**B**) Antibiotic susceptibility profile of the *lpxA* conditional mutants (*lpxA*) and their parental strains on MH agar plates supplemented with 0.125% arabinose. (**C**) Antibiotic susceptibility profile of the *accD* conditional mutants (*accD*) and their parental strains on MH agar plates supplemented with 0.5% arabinose. Values represent the mean (±standard deviation) of three independent assays. Asterisks indicate statistically significant differences with respect to the wild-type strain PAO1 or PA14 according to the ANOVA test (* *p* < 0.05). Abbreviations: Rif, rifampicin; Gm, gentamicin; Cip, ciprofloxacin; Imp, imipenem; WT, wild type.

To verify the possible impact of the deletion of *speE2* or the modulation of *lpxA* or *accD* gene expression on *P. aeruginosa* resistance to other antibiotics, we performed a Kirby–Bauer disk diffusion assay using four antibiotics belonging to different classes, i.e., rifampicin, ciprofloxacin, gentamicin, and imipenem, on plates that did not contain colistin. Since the conditional mutants require arabinose for growth, we first confirmed that arabinose does not affect the sensitivity of the wild-type strains PAO1 and PA14 to the antibiotics tested (Figure S5). Then, the assay was performed for all deletion and conditional mutants, using MH agar plates supplemented with 0.125% or 0.5% arabinose in the case of *lpxA* or *accD* conditional mutants, respectively. While the antibiotic resistance profile of all the strains tested was not affected by the *speE2* deletion, *lpxA* and *accD* conditional mutants showed increased antibiotic sensitivity under the conditions used in the assay (Figure 5). In particular, the *lpxA* conditional mutants of PAO1 showed much higher sensitivity to rifampicin, while suboptimal expression of *lpxA* slightly increased ciprofloxacin or meropenem sensitivity in PA14 derivatives. Impaired *accD* expression had a more severe effect on antibiotic susceptibility in the PAO1 genetic background, as it caused a relevant increase in rifampicin and imipenem sensitivity in all PAO1 derivatives and a slight and/or genetic background-dependent increase also in sensitivity to gentamicin and ciprofloxacin. In contrast, the antibiotic resistance defects were less marked in the *accD* conditional mutants of PA14 (Figure 5). This could explain the different effects of the *accD* conditional mutation on colistin resistance between PAO1 and PA14 (Figure 4). Indeed, it is tempting to speculate that in the PAO1 strain, the possible positive effect of reduced fatty acid biosynthesis on colistin resistance may be counteracted by major defects in the cell envelope permeability barrier that increase antibiotic susceptibility in general.

## 3. Discussion

In this study we investigated whether the genetic background or culture conditions can influence the evolution of colistin resistance in *P. aeruginosa* by performing in vitro evolution experiments with two reference strains in three different media. Our results confirmed that the acquisition of colistin resistance in *P. aeruginosa* is invariably associated with mutations in TCS genes that promote lipid A aminoarabinosylation (i.e., *pmrAB* and *phoPQ*). Other genes found to be mutated in several colistin-resistant clones are responsible for the biosynthesis of LPS, fatty acids, and norspermidine. Notably, none of these mutations were exclusively and invariably associated with a specific growth medium or strain, suggesting that colistin resistance evolution in *P. aeruginosa* is poorly affected by the genetic background and culture conditions, at least in our experimental setting. It should, however, be considered that up to 18% of *P. aeruginosa* genomes correspond to accessory genes, which are highly variable among strains and are often associated with mobile elements [44]. Thus, we cannot rule out the possibility that some of these genes could influence the evolutionary trajectories toward colistin resistance in specific isolates or under different conditions.

Mutations in genes for norspermidine and LPS biosynthesis were previously observed in colistin-resistant *P. aeruginosa* isolates [15,18,31], while mutations in genes involved in fatty acid biosynthesis have never been associated with colistin resistance in this bacterium. We demonstrated that loss-of-function mutations in norspermidine genes are beneficial for the evolution of colistin resistance in strains in which the PmrAB regulon is induced. Since norspermidine genes belong to the PmrAB regulon [31], one might hypothesize that such an effect is related to a negative impact of norspermidine (over)production on bacterial growth. However, *speE2* deletion does not affect the growth of *pmrB^M292T^* derivatives in the absence of colistin, suggesting that the deleterious effect of norspermidine (over)production occurs only in bacteria exposed to colistin. Previous studies showed that norspermidine promotes resistance to aminoglycosides by decreasing their diffusion across the outer membrane [31] and that exogenously provided norspermidine can have bactericidal activity and inhibit biofilm formation in *P. aeruginosa* [45]. Thus, it appears that this polyamine can exert different activities in *P. aeruginosa* cells, likely through different mechanisms that have not yet been elucidated. Further experiments are required to clarify the biochemical and molecular basis of norspermidine-mediated inhibition of colistin resistance in *P. aeruginosa*.

Some colistin-resistant clones carried missense mutations in *lpx* genes, in line with previous reports [15,18]. One study confirmed that the introduction of one of these mutations by allelic exchange can increase colistin resistance levels in the presence of *pmrB*-activating mutations [15]. However, the way in which *lpx* gene mutations affect colistin resistance remains unclear. We hypothesized that missense mutations in essential LPS biosynthesis genes could reduce the rates of LPS biosynthesis, leading to lower LPS levels in the outer membrane and higher resistance to the LPS-targeting antibiotic colistin. However, we found that suboptimal expression of LpxA does not increase colistin resistance, ruling out the hypothesis that reduced LPS biosynthesis alone promotes the acquisition of colistin resistance in *P. aeruginosa*.

Missense mutations associated with colistin resistance were also identified in fatty acid biosynthetic genes. Notably, metabolomic analyses highlighted a reduction in phospholipid levels in polymyxin-resistant *P. aeruginosa* isolates [25]. We investigated the possible role of fatty acid biosynthesis in colistin resistance by modulating the expression of the essential fatty acid biosynthetic gene *accD* thorough conditional mutagenesis. Impaired *accD* expression showed little effect on colistin resistance in PAO1, while it promoted resistance in PA14. Several studies have reported that the induction of colistin resistance can increase the susceptibility of Gram-negative pathogens to other antibiotics [46,47]. We therefore verified whether the mutants generated and tested for colistin resistance showed alterations in sensitivity to other antibiotics. While no alteration in the antibiotic resistance profile of ∆*speE2* mutants was observed, conditional mutants in *lpxA* and *accD* showed increased antibiotic sensitivity, which was more pronounced for *accD* conditional mutants in the PAO1 genetic background. Therefore, it is tempting to speculate that in the PAO1 strain, the possible positive effect of reduced fatty acid biosynthesis on colistin resistance could be counteracted by major defects in membrane homeostasis, thus increasing antibiotic susceptibility in general.

Overall, our findings highlighted that the inactivation of the norspermidine pathway benefits *P. aeruginosa* in acquiring high-level colistin resistance. Furthermore, we demonstrated for the first time that mutations in fatty acid biosynthesis genes can promote the evolution of colistin resistance in *P. aeruginosa*, though at the cost of increased susceptibility to other antibiotics. Whether and how frequently this occurs in polymyxin-resistant clinical isolates remain to be clarified. Recently, a biotin biosynthesis inhibitor that disrupts fatty acid metabolism was shown to restore colistin sensitivity in colistin-resistant strains of *Escherichia coli*, *Klebsiella pneumoniae*, and *Acinetobacter baumannii* and the intrinsically colistin-resistant bacterium *Serratia marcescens* [48]. Although *P. aeruginosa* was not tested in that study, our results suggest that fatty acid inhibition may not be an effective colistin-potentiating strategy against this bacterium.

## 4. Materials and Methods

### 4.1. Bacterial Strains, Plasmids, and Growth Conditions

The strains and plasmids used in this study are listed in Tables S2 and S3, respectively. Bacteria were routinely cultured in Lysogeny broth, Lennox formulation (LB; Sigma-Aldrich, St. Louis, MO, USA), for genetic manipulation, while MH broth (Difco, Detroit, MI, USA) was used for growth and antibiotic resistance assays. HS (Sigma-Aldrich) and ASM [20] were used for in vitro evolution assays. When specified, growth media were supplemented with arabinose at different concentrations as indicated. When required, antibiotics were added at the following concentrations for *E. coli* (the concentration used for *P. aeruginosa* is shown between brackets): ampicillin, 100 μg/mL; carbenicillin (500 μg/mL); nalidixic acid, 15 μg/mL, chloramphenicol, 30 μg/mL (375 μg/mL); and tetracycline, 12.5 μg/mL (100 μg/mL).

### 4.2. In Vitro Evolution Assays

Mutants with high-level colistin resistance were obtained by sequentially culturing *P. aeruginosa* strains in the presence of increasing concentrations of colistin, following a previously described protocol [16], with minor adjustments. Briefly, strains were precultured in 2 mL of MH at 37 °C and 250 rpm to the late-exponential phase and then sequentially refreshed 1:100 in 1 mL of fresh MH, ASM, or HS medium in the presence of increasing concentrations of colistin (from 0.125 to 256 µg/mL) as soon as cultures reached an optical density at 600 nm (OD_600_) > 0.5 (starting inoculum ≥ 10^7^ cells/mL). Each colistin concentration was maintained for three serial passages before doubling the concentration. Three cultures were independently evolved for each strain and culture condition. In parallel, three independent cultures for each strain and medium were cultured for the same number of passages without colistin as controls.

### 4.3. Genome Sequencing and Mutation Analysis

Genomic DNA was extracted from colistin-resistant mutants and colistin-sensitive controls using the GenElute™ Bacterial Genomic DNA Kit (Sigma-Aldrich). DNA was quantified using the Qubit 2.0 fluorometer (Thermo Fisher Scientific, Waltham, MA, USA). Paired-end libraries were prepared from 1 ng of total bacterial DNA using the Nextera XT DNA Sample Preparation kit and Nextera XT Index kit (Illumina, San Diego, CA, USA). The library concentration and average fragment size were calculated by the Qubit 2.0 fluorometer and Caliper LabChip GXI System (PerkinElmer, Waltham, MA, USA), respectively. The library concentration was normalized to 2 nM, pooled for multiplexing in equal volumes, and sequenced at 14 pM on the Illumina MiSeq platform with 300 nt paired-end reads to achieve a coverage of about 30× per base, using the MiSeq V3 flow cell at the Next Generation Sequencing (NGS) Core Facility of the Department of Cellular, Computational and Integrative Biology, University of Trento.

Bioinformatic analyses were performed using the servers of the International Centre for Genetic Engineering and Biotechnology (ICGEB-Trieste, Trieste, Italy). Fastq data were quality-checked using fastqc v. 0.11.9 [49], and adapters were removed using Trim_galore v. 0.6.7 [50]. Since the assembly of the reference strains PAO1 and PA14 gave fragmented genomes and was error-prone, we identified from GenBank the complete genome of the reference strains and compared it to the sequences of the parental strains used in our experiments as GCA_900070375.1 and GCA_900095805 for PAO1 and PA14, respectively. The SNPs between the deposited genomes and the ones used for the experiments were identified using snippy v. 3.2-dev (https://github.com/tseemann/snippy, accessed on 11 June 2025) and were added to the reference sequences to create a new reference. The annotation of the reference genome was performed using prokka v. 1.13 [51]. We identified the SNPs for each in vitro-evolved clone using the software snippy and reported them in a tabular form for subsequent analyses. All statistical analyses were conducted using R v. 4.4.2 [52].

### 4.4. Generation of Plasmids

Recombinant DNA procedures have been described elsewhere [53]. All DNA fragments for cloning were amplified by PCR with Q5 Hot Start High-Fidelity (New England Biolabs, Ipswich, MA, USA) DNA Polymerases using the genomic DNA of *P. aeruginosa* PAO1 or, when appropriate, the genomic DNA of PAO1 Col^R^ MH3 as the template. Primers and restriction enzymes used for cloning are described in Table S4. All constructs generated in this study were verified by restriction analysis and DNA sequencing and are described in Table S3.

The deletion mutagenesis constructs pDM4Δ*speE2*, pDM4Δ*lpxA*, and pDM4Δ*accD* were obtained by directionally cloning two DNA fragments of about 500 bp each, corresponding to the upstream (UP) and downstream (DOWN) regions of the coding sequence of each gene of interest, into pBluescript II KS+ (Table S3), followed by DNA sequencing and subcloning of the entire insert encompassing the upstream and downstream regions into the suicide vector pDM4 [54]. The allele replacement construct pDM4*pmrB^M292T^* was generated by cloning the mutated *pmrB* allele of PAO1 Col^R^ MH3, previously amplified by PCR, into pBS, followed by DNA sequencing to check the presence of the desired mutation and subcloning into pDM4.

For the generation of the constructs for arabinose-dependent gene expression, PCR-amplified DNA fragments corresponding to the coding sequence of each gene of interest (*lpxA* or *accD*) were directionally cloned into mini-CTX1 *araC*-P_BAD_ downstream of the arabinose-dependent regulatory element *araC*-P_BAD_ [40].

### 4.5. Generation of Deletion, Allele Replacement, and Conditional Mutants

The pDM4 derivatives for the deletion of the genes of interest (Table S3) were individually transferred into *P. aeruginosa* strains by conjugation. Transconjugants were selected on LB agar plates containing 15 μg/mL nalidixic acid and 375 μg/mL chloramphenicol. Deletion mutants were obtained through homologous recombination and sucrose-based selection, as previously described [55]. The deletion of *speE2*, *lpxA*, or *accD* was confirmed by PCR, using the primers listed in Table S4. In the case of *pmrB* allele replacement, clones obtained after sucrose-based selection were screened on plates containing 8 μg/mL colistin to identify colistin-resistant clones that acquired the *pmrB*^M292T^ allele. The presence of the *pmrB*^M292T^ allele in selected mutants was confirmed by PCR followed by DNA sequencing, using the primers listed in Table S4.

To obtain *P. aeruginosa* conditional mutants, the *lpxA* or *accD* coding sequence under the control of the arabinose-dependent regulatory element and their respective mini-CTX1 *araC*-P_BAD_ were integrated into the *attB* neutral site of the *P. aeruginosa* chromosome, and the mini-CTX1 plasmid backbone was excised by Flp-mediated site-specific recombination as previously described [55,56]. Then, in-frame deletion mutagenesis of the endogenous copy of *lpxA* or *accD* was conducted under permissive condition (i.e., growth in the presence of 0.5% arabinose) using the construct pDM4*lpxA* or pDM4*accD* and the procedure described above.

### 4.6. Growth and Minimum Inhibitory Concentration Assays

For in vitro-evolved strains, the colistin MIC was determined through the broth microdilution method in MH. Strains were cultured at 37 °C for 8 h and then refreshed at about 5 × 10^5^ cells/mL in the presence of increasing concentrations of colistin (up to 512 μg/mL). The MIC was visually recorded after 20 h at 37 °C. MIC values represent the mode of at least three independent experiments.

For the validation of the conditional mutants, strains were cultured at 37 °C for 8 h in MH supplemented with 0.5% arabinose. Bacteria were refreshed at OD_600_ = 0.002 in 96-well polystyrene plates and cultured in MH in the presence or absence of different concentrations of arabinose (from 0.03 to 0.5%) for 20 h at 37 °C in a Tecan Spark 10M microtiter plate reader. Growth was measured over time as the OD_600_ of the bacterial cultures.

For growth assays in the presence of different concentrations of colistin, strains were cultured at 37 °C for 8 h in MH, supplemented with 0.5% arabinose in the case of conditional mutants. Bacteria were refreshed at about 5 × 10^5^ cells/mL in MH supplemented or not with a suboptimal concentration of arabinose (as indicated in the text and in the figure legends) and containing increasing concentrations of colistin. The MIC was defined as the lowest concentration of the antibiotic for which no growth was observed after 20 or 45 h of incubation at 37 °C in a Tecan Spark 10M microtiter plate reader.

### 4.7. Kirby–Bauer Disk Diffusion Assay

Bacteria were cultured until the mid-exponential phase in MH, supplemented with 0.5% arabinose in the case of conditional mutants. Bacterial cells were harvested by centrifugation, resuspended in saline at 0.5 McFarland Standard, and swabbed onto MH agar plates, supplemented with different arabinose concentrations as indicated. Disks containing rifampicin (5 µg), gentamicin (10 μg), ciprofloxacin (5 µg), or imipenem (10 µg) (Becton Dickinson, Franklin Lakes, NJ, USA) were placed on the surface of the inoculated plates, and growth inhibition halo diameters were measured after 24 h incubation at 37 °C.

### 4.8. Statistical Analysis

Statistical analysis was performed with the software GraphPad Instat v. 3.0, using the One-Way Analysis of Variance (ANOVA) followed by a Tukey multiple comparison test.

## Figures and Tables

**Table 1:** Genes in which mutations associated with colistin resistance were repeatedly identified in colistin-resistant clones ^1^.

Strain	Medium	TCSs Controlling Lipid A Aminoarabinosylation				LPS Synthesis and Modification						Norspermidine Synthesis		Fatty Acid Synthesis			
		*pmrA*	*pmrB*	*phoP*	*phoQ*	*lpxA*	*lpxC*	*lpxD*	*lpxT*	*lpxO2*	*galU*	*speD2*	*speE2*	*accD*	*acpP*	*fabB*	*fabY*
PAO1	MH		3					2									
	ASM	1	2						1	1	1		3				
	HS		2		1					1			2		1		
PA14	MH		1	1	2					1		1					
	ASM		3			1						2	1	1			
	HS	1			2	1	2			1		1				1	1

^1^ Numbers refer to the number of clones showing at least one mutation in the reported gene for each strain and medium. Only genes or gene categories found to be mutated in at least three colistin-resistant clones are included in the table. Additional mutations are reported in Table S1.

## Data Availability

The main data are included in the manuscript and Supplementary Materials. Sequencing reads have been deposited in the NCBI Sequence Read Archive (SRA) and are publicly accessible under BioProject accession number PRJNA1272331. Additional data are available upon request.

## Supplementary Materials

The following supporting information can be downloaded at https://www.mdpi.com/article/10.3390/antibiotics14060601/s1, Table S1: Mutations specifically identified in colistin-resistant clones; Table S2: Bacterial strains used in this study; Table S3: Plasmids used in this study; Table S4: Primers used in this study; Figure S1: In vitro evolution of colistin-resistant clones of *P. aeruginosa* PAO1 and PA14 in MH, ASM, or HS; Figure S2: Colistin MIC of *P. aeruginosa* PAO1 and PA14 and their P*rpsA*::*arn* or *pmrB^M292T^* derivatives; Figure S3: Validation of the arabinose-dependent *lpxA* and *accD* conditional mutants; Figure S4: Growth curves and colistin MIC of *lpxA* conditional mutants cultured in the presence of 0.0625% arabinose; Figure S5: Antibiotic sensitivity profile of the wild-type strains PAO1 and PA14 in the presence or absence of 0.5% arabinose. References [57,58,59,60] are cited in the Supplementary Materials.

## Abbreviations

The following abbreviations are used in this manuscript:

ASM	Artificial sputum medium
*arn*	*arnBCADTEF-ugd*
HS	Heat-inactivated human serum
L-Ara4N	4-amino-4-deoxy-L-arabinose
LB	Lysogeny broth
LPS	Lipopolysaccharide
MH	Mueller–Hinton
NGS	Next Generation Sequencing
PEtN	Phosphoethanolamine
TCS	Two-component system
