# Supplementary material for: The Genetic Background and Culture Medium Only Marginally Affect the In Vitro Evolution of Pseudomonas aeruginosa Toward Colistin Resistance
Source: Antibiotics (Basel). 2025 Jun 13;14(6):601. doi: 10.3390/antibiotics14060601 (PMC12189927; doi:10.3390/antibiotics14060601)
Supplement: Supplementary file 1 [file antibiotics-14-00601-s001.zip › Supplementary material_revised.pdf]

The Genetic Background and Culture Medium Only Marginally Affect the In Vitro Evolution of *Pseudomonas aeruginosa* Toward Colistin Resistance

Matteo Cervoni, Antonio Maria Ferriero, Alessandra Lo Sciuto, Francesca Guidi, Naida Babić Jordamović, Silvano Piazza, Olivier Jousson, Alfonso Esposito, Francesco Imperi

SUPPLEMENTARY MATERIAL

Table S1. Mutations specifically identified in colistin resistant clones.

Table S2. Bacterial strains used in this study.

Table S3. Plasmids used in this study.

Table S4. Primers used in this study.

Figure S1. *In vitro* evolution of colistin resistant clones of *P. aeruginosa* PAO1 and PA14 in MH, ASM, or HS.

Figure S2. Colistin MIC of *P. aeruginosa* PAO1 and PA14 and their P*rpsA::arn* or *pmrB*^{M292T} derivatives.

Figure S3. Validation of the arabinose-dependent *lpxA* and *accD* conditional mutants.

Figure S4. Growth curves and colistin MIC of *lpxA* conditional mutants cultured in the presence of 0.0625% arabinose.

Figure S5. Antibiotic sensitivity profile of the wild type strains PAO1 and PA14 in the presence or absence of 0.5% arabinose.

Table S2. Bacterial strains used in this study.

Strain	Genotype and/or relevant characteristics	Reference/source
E. coli		
S17.1*λpir*	*thi pro hsdR hsdM*$^+$ *recA* RP4-2-Tc::Mu-Km::Tn7*λpir*, Gm^R	[57]
SM10*λpir*	*thi-1 thr leu tonA lacY supE recA*::RP4-2- Tc::Mu Km^R	[58]
P. aeruginosa		
PAO1	Reference strain, wild type	American Type Culture Collection
PA14	Reference strain, wild type	[59]
PAO1 Col^R MH1	Colistin resistant derivative of PAO1 obtained through *in vitro* evolution in MH in the presence of increasing colistin concentrations	This work
PAO1 Col^R MH2	Colistin resistant derivative of PAO1 obtained through *in vitro* evolution in MH in the presence of increasing colistin concentrations	This work
PAO1 Col^R MH3	Colistin resistant derivative of PAO1 obtained through *in vitro* evolution in MH in the presence of increasing colistin concentrations	This work
PAO1 Col^R ASM1	Colistin resistant derivative of PAO1 obtained through *in vitro* evolution in ASM in the presence of increasing colistin concentrations	This work
PAO1 Col^R ASM2	Colistin resistant derivative of PAO1 obtained through *in vitro* evolution in ASM in the presence of increasing colistin concentrations	This work
PAO1 Col^R ASM3	Colistin resistant derivative of PAO1 obtained through *in vitro* evolution in ASM in the presence of increasing colistin concentrations	This work
PAO1 Col^R HS1	Colistin resistant derivative of PAO1 obtained through *in vitro* evolution in HS in the presence of increasing colistin concentrations	This work
PAO1 Col^R HS2	Colistin resistant derivative of PAO1 obtained through *in vitro* evolution in HS in the presence of increasing colistin concentrations	This work
PAO1 Col^R HS3	Colistin resistant derivative of PAO1 obtained through *in vitro* evolution in HS in the presence of increasing colistin concentrations	This work
PA14 Col^R MH1	Colistin resistant derivative of PA14 obtained through *in vitro* evolution in MH in the presence of increasing colistin concentrations	This work
PA14 Col^R MH2	Colistin resistant derivative of PA14 obtained through *in vitro* evolution in MH in the presence of increasing colistin concentrations	This work
PA14 Col^R MH3	Colistin resistant derivative of PA14 obtained through *in vitro* evolution in MH in the presence of increasing colistin concentrations	This work
PA14 Col^R ASM1	Colistin resistant derivative of PA14 obtained through *in vitro* evolution in ASM in the presence of increasing colistin concentrations	This work
PA14 Col^R ASM2	Colistin resistant derivative of PA14 obtained through *in vitro* evolution in ASM in the presence of increasing colistin concentrations	This work
PA14 Col^R ASM3	Colistin resistant derivative of PA14 obtained through *in vitro* evolution in ASM in the presence of increasing colistin concentrations	This work
PA14 Col^R HS1	Colistin resistant derivative of PA14 obtained through *in vitro* evolution in HS in the presence of increasing colistin concentrations	This work
PA14 Col^R HS2	Colistin resistant derivative of PA14 obtained through *in vitro* evolution in HS in the presence of increasing colistin concentrations	This work
PA14 Col^R HS3	Colistin resistant derivative of PA14 obtained through *in vitro* evolution in HS in the presence of increasing colistin concentrations	This work
PAO1 Col^S MH1	Colistin sensitive derivative of PAO1 obtained through *in vitro* evolution in MH in the absence of colistin	This work
PAO1 Col^S MH2	Colistin sensitive derivative of PAO1 obtained through *in vitro* evolution in MH in the absence of colistin	This work

PAO1 Col^S MH3	Colistin sensitive derivative of PAO1 obtained through *in vitro* evolution in MH in the absence of colistin	This work
PAO1 Col^S ASM1	Colistin sensitive derivative of PAO1 obtained through *in vitro* evolution in ASM in the absence of colistin	This work
PAO1 Col^S ASM2	Colistin sensitive derivative of PAO1 obtained through *in vitro* evolution in ASM in the absence of colistin	This work
PAO1 Col^S ASM3	Colistin sensitive derivative of PAO1 obtained through *in vitro* evolution in ASM in the absence of colistin	This work
PAO1 Col^S HS1	Colistin sensitive derivative of PAO1 obtained through *in vitro* evolution in HS in the absence of colistin	This work
PAO1 Col^S HS2	Colistin sensitive derivative of PAO1 obtained through *in vitro* evolution in HS in the absence of colistin	This work
PAO1 Col^S HS3	Colistin sensitive derivative of PAO1 obtained through *in vitro* evolution in HS in the absence of colistin	This work
PA14 Col^S MH1	Colistin sensitive derivative of PA14 obtained through *in vitro* evolution in MH in the absence of colistin	This work
PA14 Col^S MH2	Colistin sensitive derivative of PA14 obtained through *in vitro* evolution in MH in the absence of colistin	This work
PA14 Col^S MH3	Colistin sensitive derivative of PA14 obtained through *in vitro* evolution in MH in the absence of colistin	This work
PA14 Col^S ASM1	Colistin sensitive derivative of PA14 obtained through *in vitro* evolution in ASM in the absence of colistin	This work
PA14 Col^S ASM2	Colistin sensitive derivative of PA14 obtained through *in vitro* evolution in ASM in the absence of colistin	This work
PA14 Col^S ASM3	Colistin sensitive derivative of PA14 obtained through *in vitro* evolution in ASM in the absence of colistin	This work
PA14 Col^S HS1	Colistin sensitive derivative of PA14 obtained through *in vitro* evolution in HS in the absence of colistin	This work
PA14 Col^S HS2	Colistin sensitive derivative of PA14 obtained through *in vitro* evolution in HS in the absence of colistin	This work
PA14 Col^S HS3	Colistin sensitive derivative of PA14 obtained through *in vitro* evolution in HS in the absence of colistin	This work
PAO1 P*rpsA::arn*	PAO1 derivative in which the promoter of the *arn* operon is replaced by the promoter of the housekeeping gene *rpsA*	[20]
PA14 P*rpsA::arn*	PA14 derivative in which the promoter of the *arn* operon is replaced by the promoter of the housekeeping gene *rpsA*	[20]
PAO1 $pmrB^{M292T}$	PAO1 derivative in which the wild-type allele *pmrB* is replaced by the mutated allele $pmrB^{M292T}$ obtained from the strain PAO1 Col^R MH3	This work
PA14 $pmrB^{M292T}$	PA14 derivative in which the wild-type allele *pmrB* is replaced by the mutated allele $pmrB^{M292T}$ obtained from the strain PAO1 Col^R MH3	This work
PAO1 Δ*speE2*	PAO1 derivative with an in-frame deletion of the *speE2* coding sequence	This work
PA14 Δ*speE2*	PA14 derivative with an in-frame deletion of the *speE2* coding sequence	This work
PAO1 P*rpsA::arn* Δ*speE2*	PAO1 P*rpsA::arn* derivative with an in-frame deletion of the *speE2* coding sequence	This work
PA14 P*rpsA::arn* Δ*speE2*	PA14 P*rpsA::arn* derivative with an in-frame deletion of the *speE2* coding sequence	This work
PAO1 $pmrB^{M292T}$ Δ*speE2*	PAO1 $pmrB^{M292T}$ derivative with an in-frame deletion of the *speE2* coding sequence	This work
PA14 $pmrB^{M292T}$ Δ*speE2*	PA14 $pmrB^{M292T}$ derivative with an in-frame deletion of the *speE2* coding sequence	This work

PAO1 *araC* P_{BAD}::*lpxA* Δ*lpxA*	*lpxA* conditional mutant of PAO1, deleted of the *lpxA* coding sequence and carrying an arabinose-dependent copy of *lpxA* in a neutral chromosomal site	This work
PA14 *araC* P_{BAD}::*lpxA* Δ*lpxA*	*lpxA* conditional mutant of PA14, deleted of the *lpxA* coding sequence and carrying an arabinose-dependent copy of *lpxA* in a neutral chromosomal site	This work
PAO1 P*rpsA::arn araC* P_{BAD}::*lpxA* Δ*lpxA*	*lpxA* conditional mutant of PAO1 P*rpsA::arn*, deleted of the *lpxA* coding sequence and carrying an arabinose-dependent copy of *lpxA* in a neutral chromosomal site	This work
PA14 P*rpsA::arn araC* P_{BAD}::*lpxA* Δ*lpxA*	*lpxA* conditional mutant of PA14 P*rpsA::arn*, deleted of the *lpxA* coding sequence and carrying an arabinose-dependent copy of *lpxA* in a neutral chromosomal site	This work
PAO1 *pmrB*^{M292T} *araC* P_{BAD}::*lpxA* Δ*lpxA*	*lpxA* conditional mutant of PAO1 *pmrB*^{M292T}, deleted of the *lpxA* coding sequence and carrying an arabinose-dependent copy of *lpxA* in a neutral chromosomal site	This work
PA14 *pmrB*^{M292T} *araC* P_{BAD}::*lpxA* Δ*lpxA*	*lpxA* conditional mutant of PA14 *pmrB*^{M292T}, deleted of the *lpxA* coding sequence and carrying an arabinose-dependent copy of *lpxA* in a neutral chromosomal site	This work
PAO1 *araC* P_{BAD}::*accD* Δ*accD*	*accD* conditional mutant of PAO1, deleted of the *accD* coding sequence and carrying an arabinose-dependent copy of *accD* in a neutral chromosomal site	This work
PA14 *araC* P_{BAD}::*accD* Δ*accD*	*accD* conditional mutant of PA14, deleted of the *accD* coding sequence and carrying an arabinose-dependent copy of *accD* in a neutral chromosomal site	This work
PAO1 P*rpsA::arn araC* P_{BAD}::*accD* Δ*accD*	*accD* conditional mutant of PAO1 P*rpsA::arn*, deleted of the *accD* coding sequence and carrying an arabinose-dependent copy of *accD* in a neutral chromosomal site	This work
PA14 P*rpsA::arn araC* P_{BAD}::*accD* Δ*accD*	*accD* conditional mutant of PA14 P*rpsA::arn*, deleted of the *accD* coding sequence and carrying an arabinose-dependent copy of *accD* in a neutral chromosomal site	This work
PAO1 *pmrB*^{M292T} *araC* P_{BAD}::*accD* Δ*accD*	*accD* conditional mutant of PAO1 *pmrB*^{M292T}, deleted of the *accD* coding sequence and carrying an arabinose-dependent copy of *accD* in a neutral chromosomal site	This work
PA14 *pmrB*^{M292T} *araC* P_{BAD}::*accD* Δ*accD*	*accD* conditional mutant of PA14 *pmrB*^{M292T}, deleted of the *accD* coding sequence and carrying an arabinose-dependent copy of *accD* in a neutral chromosomal site	This work

Table S3. Plasmids used in this study.

Plasmid	Relevant characteristics	Reference/source
pBluescript II (pBS)	Cloning and sequencing vector; ColE1 replicon; Ap^R	Stratagene
pBS*pmrB*^{M292T}	pBS derivative carrying a 1,376 bp DNA fragment encompassing the *pmrB*^{M292T} allele of PAO1 Col^R MH3	This work
pBS*speE2*	pBS derivative carrying a 991bp DNA fragment encompassing the UP and DOWN fragments of the *speE2* coding sequence	This work
pBS*lpxA*	pBS derivative carrying a 989 bp DNA fragment encompassing the UP and DOWN fragments of the *lpxA* coding sequence	This work
pBS*accD*	pBS derivative carrying a 999 bp DNA fragment encompassing the UP and DOWN fragments of the *accD* coding sequence	This work
pDM4	Suicide vector in *P. aeruginosa*; *sacB*, *oriR6K*; Cm^R	[54]
pDM4*pmrB*^{M292T}	pDM4 derivative carrying the *pmrB*^{M292T} fragment excised from pBS*pmrB*^{M292T}, used for allele replacement	This work
pDM4Δ*speE2*	pDM4 derivative carrying the UP and DOWN fragments of *speE2*, used for the in-frame deletion of the *speE2* coding sequence	This work
pDM4Δ*lpxA*	pDM4 derivative carrying the UP and DOWN fragments of *lpxA*, used for the in-frame deletion of the *lpxA* coding sequence	This work
pDM4Δ*accD*	pDM4 derivative carrying the UP and DOWN fragments of *accD*, used for the in-frame deletion of the *accD* coding sequence	This work
mini-CTX1	Self-proficient integration vector with *tet*, Ω-FRT-*attP*-MCS, *ori*, *int*, and *oriT*; Tc^R	[56]
mini-CTX1 *araC* P_{BAD}	mini-CTX1 derivative carrying the *araC* P_{BAD} regulatory element; Tc^R	[40]
mini-CTX1 *araC* P_{BAD} *lpxA*	mini-CTX1 *araC* P_{BAD} derivative carrying the coding sequence of *lpxA* downstream of P_{BAD}	This work
mini-CTX1 *araC* P_{BAD} *accD*	mini-CTX1 *araC* P_{BAD} derivative carrying the coding sequence of *accD* downstream of P_{BAD}	This work
pFLP2	Vector for FLP expression and site specific FRT-mediated recombination; *sacB*, Ap^R/Cb^R	[60]

Table S4. Primers used in this study.[1]

Primer	Sequence (5'-3')[2]	Restriction sites[3]	Application
*pmrB*_FW2	acgcgtcgaCGGCTTCGTGCTGTGCTG	SalI	Generation of pDM4*pmrB*^{M292T} and DNA sequencing
*pmrB*_RV2	gctctAGATATGTGACCGCCCGCT	XbaI	
*speE2*_mut_UP_FW	cccCTCGAGCATGGACCGCGG	XhoI	Generation of pDM4Δ*speE2*
*speE2*_mut_UP_RV	cgggatcCGTGGTACAGCGACTCATC	BamHI	
*speE2*_mut_DOWN_FW	cgggatCCTGAAGATGTCCGCCGC	BamHI	
*speE2*_mut_DOWN_RV	gctctagAGTTCGACATCGGCCTGGG	XbaI	
*lpxA*_ara_FW	cgcaagctTATGAGTTTGATCGATCCTCG	HindIII	Generation of a mini-CTX1 *araC* P_{BAD} *lpxA*
*lpxA*_ara_RV	cggaatTCGCCGGCGACCAGGGC	EcoRI	
*lpxA*_mut_UP_FW	ccccctCGAGATTCGCGAATACCTGC	XhoI	Generation of pDM4Δ*lpxA*
*lpxA*_mut_UP_RV	cgggatCCACGGGCCCACCTGGA	BamHI	
*lpxA*_mut_DOWN_FW	ccggATCCAGAGCGCAACCCGC	BamHI	
*lpxA*_mut_DOWN_RV	gctcTAGAAACGTGCTTCGAAGGG	XbaI	
*accD*_ara_FW	tcccccggGCATGAGCAACTGGCTGGT	SmaI	Generation of a mini-CTX1 *araC* P_{BAD} *accD*
*accD*_ara_RV	cggaatTCGGATGGAGTTGTTCGAGA	EcoRI	
*accD*_mut_UP_FW	cccctcgaGACCTGGGCAAACCCCTG	XhoI	Generation of pDM4Δ*accD*
*accD*_mut_UP_RV	cgggatcCGCATGATGGAAGGGATCAG	BamHI	
*accD*_mut_DOWN_FW	ccggatCCTGGCCAATCTGCTGTCG	BamHI	
*accD*_mut_DOWN_RV	gctctaGACCGCATCCAGGCGCC	XbaI	
*speE2*_RT_FW	GGAGCTGGTGGAACTGTGTC		Validation of deletion mutants
*speE2*_RT_RV	CTGGCGGGTATAGAGAGCCT		
*lpxA*_RT_FW	GAAATACAAGGGCGAGCCGA		
*lpxA*_RT_RV	AGGATGCAATGGTTGCCGAT		
*accD*_RT_FW	GAAGGCCTGTGGCACAAATG		
*accD*_RT_RV	CTTCTTGCTGTCGCGGAATTT		
M13_FW	GTTTTCCCAGTCACGAC		Sequencing of fragments cloned into pBS or mini-CTX1 *araC* P_{BAD}
M13_RV	AACAGCTATGACCATG		
pBAD_primer_FW	CATAAGATTAGCGGATCCTAC		
phoPQ seq check_FW	AGATGACCCCGCATGGCG		PCR amplification and sequencing of *phoPQ*
phoPQ seq check_RV	AGCCGAACAGACTTCAGCG		
phoPQ int seq	CGACGAGGAGCGCGACG		

[1] All preparative PCRs were performed using the genomic DNA of *P. aeruginosa* PAO1 as the template, except for the *pmrB*^{M292T} fragment that was amplified from the genomic DNA of PAO1 Col^{R} MH3.
[2] Lowercase letters indicate the region of the primer that does not anneal to the template.
[3] The restriction site used for cloning is underlined in the primer sequence.

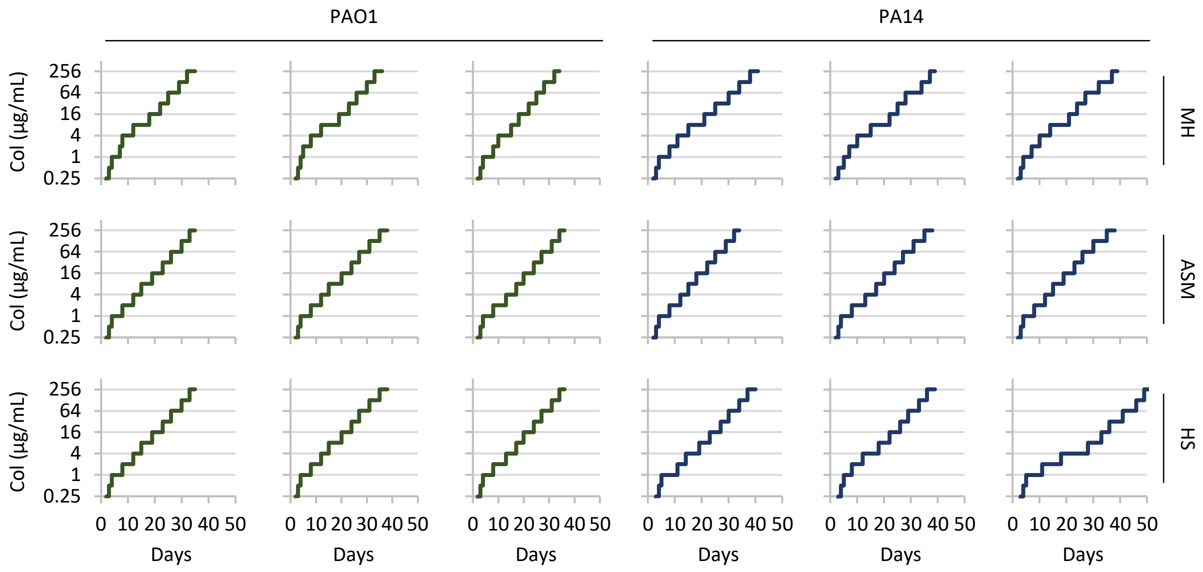

Figure S1. *In vitro* evolution of three independent clones of *P. aeruginosa* PAO1 or PA14 in MH, ASM, or HS, through serial passages in the presence of increasing colistin concentrations (up to 256 µg/mL).

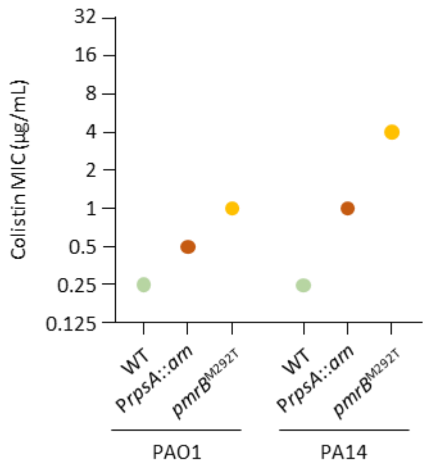

Figure S2. Colistin MIC of the *P. aeruginosa* strains PAO1, PAO1 P*rpsA::arn*, PAO1 *pmrB*^{M292T}, PA14, PA14 P*rpsA::arn*, and PA14 *pmrB*^{M292T} after 20 h of growth in MH. Values correspond to the mode of at least three independent experiments.

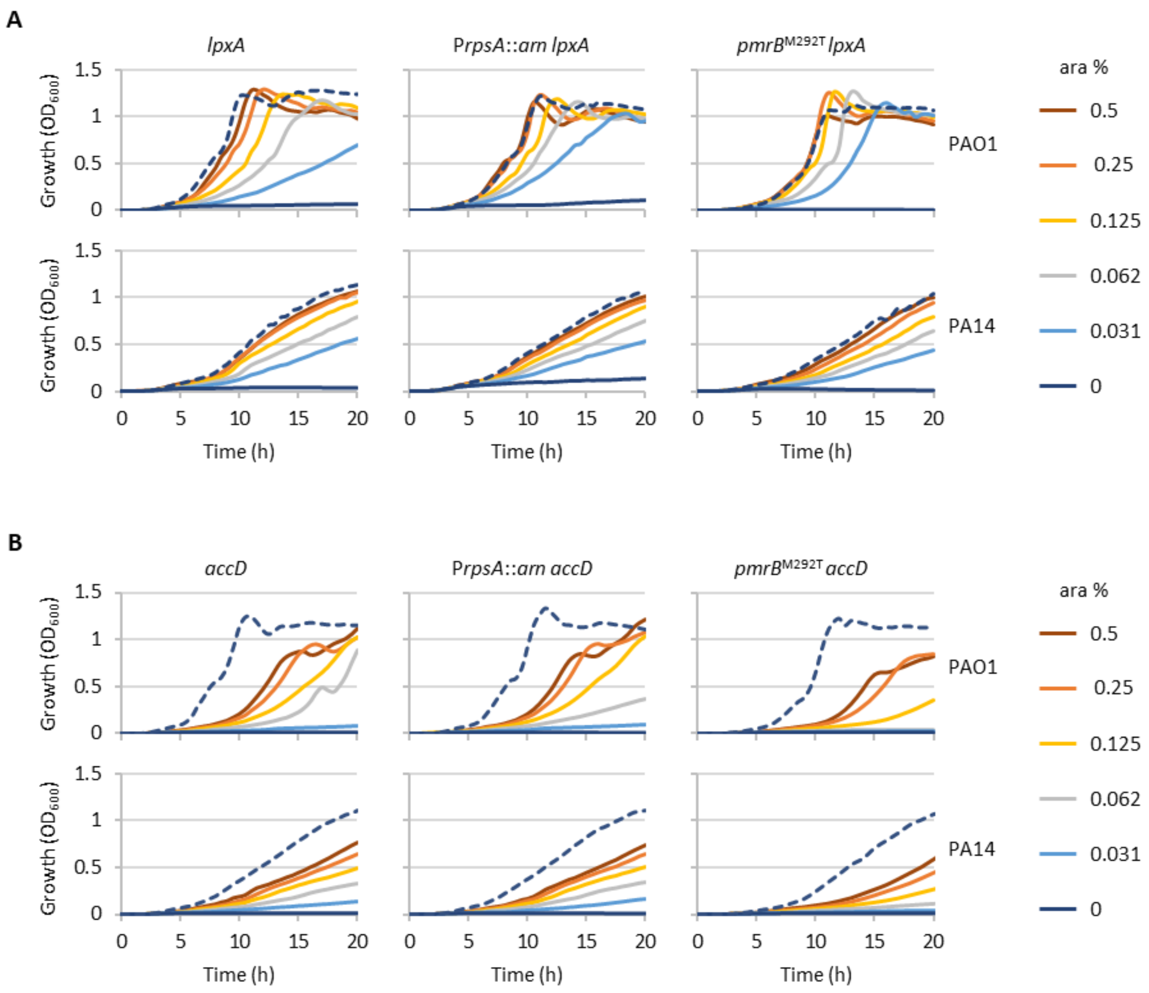

Figure S3. Validation of the *lpxA* and *accD* conditional mutants. (**A**) Growth curves of the *araC*-PBAD::*lpxA* Δ*lpxA* conditional mutants in the parental strains PAO1 and PA14 (*lpxA*), the *PrpsA::arn* derivatives (*PrpsA::arn lpxA*), or the strains carrying the *pmrB*^{M292T} allele (*pmrB*^{M292T} *lpxA*) in MH in the presence of increasing concentrations of arabinose (Ara). (**B**) Growth curves of the *accD* conditional mutants in the parental strains PAO1 and PA14 (*accD*), the *PrpsA::arn* derivatives (*PrpsA::arn accD*), or the strains carrying the *pmrB*^{M292T} allele (*pmrB*^{M292T} *accD*) in MH in the presence of increasing concentrations of arabinose (ara). Growth curves are representative of three independent experiments giving similar results. Dashed lines represent the growth curves of the corresponding parental strains in MH.

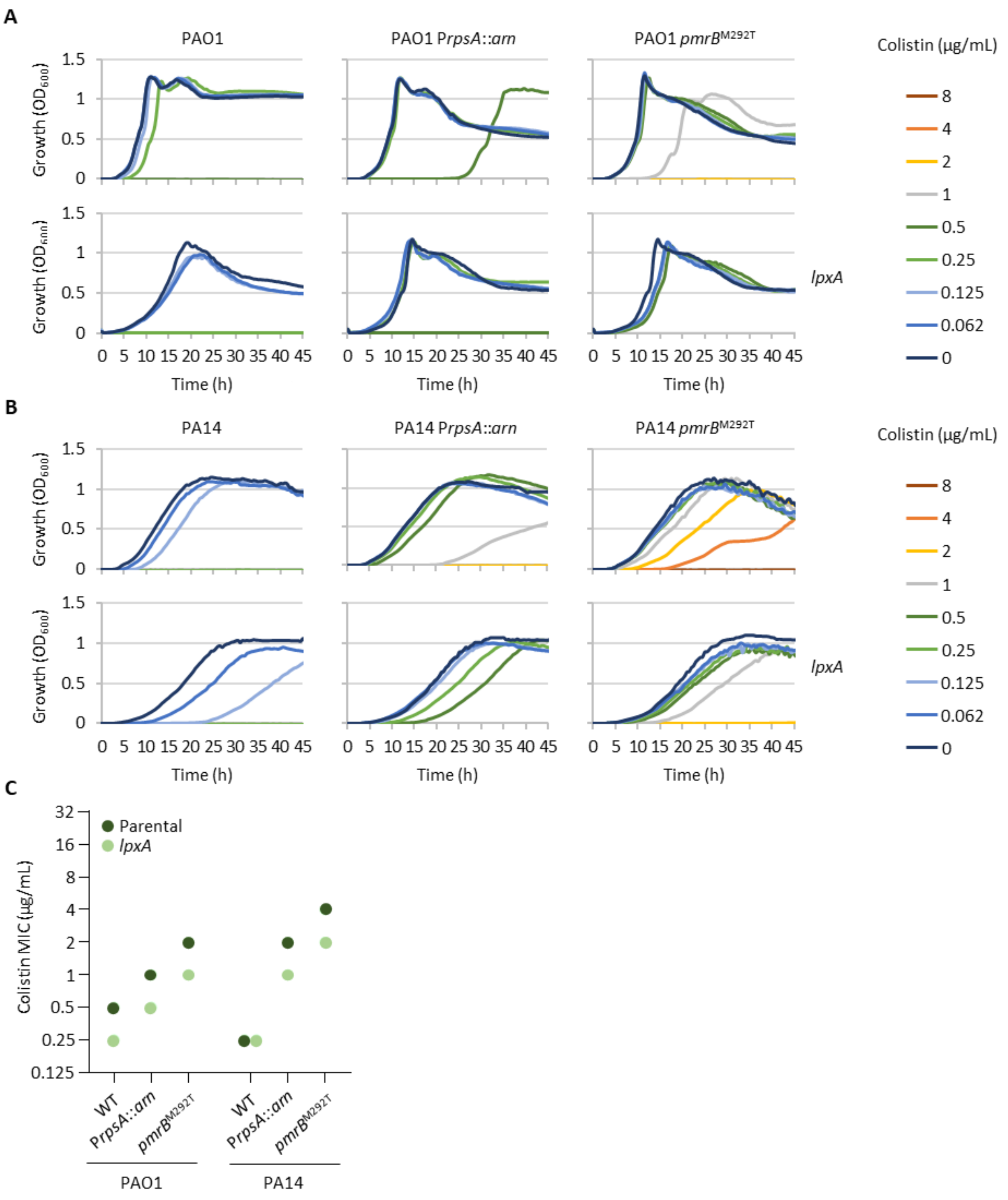

Figure S4. (A) Growth curves of PAO1, PAO1 P*rpsA::arn*, PAO1 *pmrB*^{M292T}, PAO1 *araC*-P$_{BAD}$::*lpxA* Δ*lpxA*, PAO1 *PrpsA::arn araC*-P$_{BAD}$::*lpxA* Δ*lpxA*, PAO1 *pmrB*^{M292T} *araC*-P$_{BAD}$::*lpxA* Δ*lpxA* in MH supplemented with 0.0625% arabinose and increasing colistin concentrations. **(B)** Growth curves of PA14, PA14 P*rpsA::arn*, PA14 *pmrB*^{M292T}, PA14 *araC*-P$_{BAD}$::*lpxA* Δ*lpxA*, PA14 *PrpsA::arn araC*-P$_{BAD}$::*lpxA* Δ*lpxA* and PA14 *pmrB*^{M292T} *araC*-P$_{BAD}$::*lpxA* Δ*lpxA* in MH supplemented with 0.0625% arabinose and increasing colistin concentrations. **(C)** Colistin MIC for the above-mentioned strains after 20 h of growth in MH supplemented with 0.0625% arabinose. Growth curves are representative of at least three independent experiments giving similar results. MIC values correspond to the mode of at least three independent experiments.

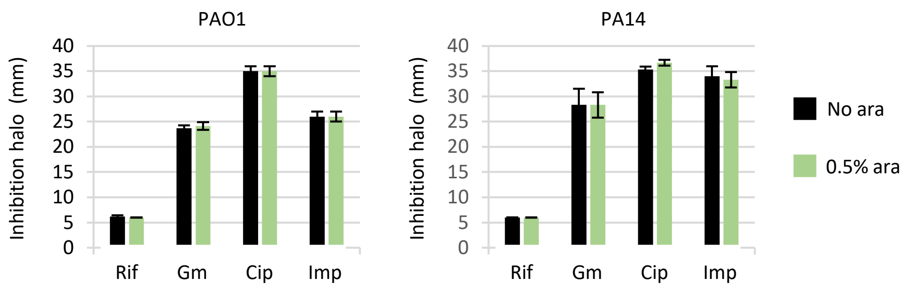

Figure S5. Kirby-Bauer disc diffusion assay for the wild type strains PAO1 and PA14 cultured on MH agar plates containing or not 0.5% arabinose (ara). Values are the mean (± standard deviation) of three independent experiments. Abbreviations: Rif, rifampicin; Gm, gentamicin; Cip, ciprofloxacin; Imp, imipenem.
